# Responsiveness of ThymoDream^®^ as a natural modulator of the circadian axis: a comparative RCT with melatonin supplementation employing melatonin profiling and actigraphy

**DOI:** 10.3389/frsle.2026.1879110

**Published:** 2026-09-11

**Authors:** C. S. Harshith, Thomas V. Jestin, Das S. Syam, Prabhakaran Prathibha, Nithyanandam Allimuthu, N. Abhirami, A. Radhika, I. M. Krishnakumar, Janeesh Plakkal Ayyappan

**Affiliations:** 1 BGS Global Institute of Medical Sciences, Bangalore, Karnataka, India; 2 Leads Clinical Research and Bio Services Pvt. Ltd, Bangalore, Karnataka, India; 3 Akay Natural Ingredients Private Limited, Kochi, Kerala, India; 4 Tamil Nadu Government Multi Super Specialty Hospital, Chennai, Tamil Nadu, India; 5 Translational Nanomedicine and Lifestyle Disease Research Laboratory, Department of Biochemistry, University of Kerala, Thiruvananthapuram, Kerala, India; 6 Department of Biochemistry, University of Kerala, Thiruvananthapuram, Kerala, India; 7 Centre for Advanced Cancer Research, Department of Biochemistry, University of Kerala, Thiruvananthapuram, Kerala, India; 8 Department of Biochemistry, Government Arts and Science College, Kulathoor, Thiruvananthapuram, Kerala, India

**Keywords:** circadian rhythm, cortisol, dim light melatonin onset (DLMO), melatonin, *Nigella sativa*, sleep, stress, thymoquinone

## Abstract

**Introduction:**

Non-restorative sleep is frequently associated with disturbances in circadian regulation, impaired sleep quality, and reduced daytime functioning.

**Methods:**

This randomized, double-blind, active-controlled, cross over study compared black cumin oil extract (TD; 200 mg) with synthetic melatonin (s-Mel; 5 mg/day) in 24 individuals with non-restorative sleep. Participants received each intervention for 7 nights separated by a 7-day washout period. Plasma melatonin was measured at fixed nighttime intervals under dim light to determine dim-light melatonin onset (DLMO), while sleep parameters were assessed using actigraphy. DLMO serves as an important physiological marker of circadian timing. Daytime sleepiness was evaluated with the Epworth Sleepiness Scale (ESS).

**Results:**

Both interventions significantly increased nocturnal plasma melatonin concentration under dim-light conditions (*P* < 0.001). TD was associated with an earlier attainment of the predefined plasma melatonin threshold of 10 pg/mL.

**Discussion:**

The interpretation of the corresponding threshold timing in the s-Mel arm was limited due to exogenous melatonin administration. s-Mel induced a rapid early rise in melatonin followed by a sharp decline, whereas TD demonstrated a more sustained nocturnal profile. Actigraphy outcomes were generally comparable; sleep latency and total sleep time improved more with s-Mel, while TD significantly reduced cortisol levels and daytime sleepiness. Overall, TD showed earlier threshold attainment, cortisol reduction, and improved daytime alertness, supporting its potential as a natural alternative for circadian disruption and non-restorative sleep.

**Clinical trial registration:**

https://ctri.nic.in/Clinicaltrials/pmaindet2.php?EncHid=MTExNTY4&Enc=&userName= CTRI/2024/11/077454 dated 28/11/2024.

## 1 Introduction

Stress, defined as the body's physiological or psychological response to external stimuli, has been shown to disrupt sleep, and consequently the circadian rhythm (CR). The CR represents the body's intrinsic molecular clock, programmed to synchronize physiological, mental, and behavioral processes within the 24-h light-dark cycle. Disturbances in CR and sleep disorders can worsen physiological function across metabolic, immune, and neuroendocrine systems (Hertenstein et al., 2019; Palagini et al., 2024), leading to health issues such as metabolic disorders, obesity, depression, mood swings, reproductive health issues, cognitive impairment, and autonomic imbalances (Hertenstein et al., 2019; Palagini et al., 2024; Walker et al., 2020; Zhong et al., 2022). Accordingly, the “stress–sleep–circadian axis” has emerged as a critical determinant of overall health and disease susceptibility (Foster, 2020). According to a recent report, sleep disorder has become one of the most common health issues with 16% people affected globally: 64% in USA, 20% in Europe and around 40% in Asia (Benjafield et al., 2025). A study conducted among 300,000 participants from 131 countries, stress was considered as one of the key factors for insomnia affecting nearly 35% of the population worldwide (Smith and Wesselbaum, 2025). These data highlight the importance of understanding the biological mechanisms that regulate stress and sleep, particularly the role of melatonin in maintaining healthy circadian rhythm.

Melatonin (N-acetyl-5-methoxytryptamine), primarily produced by the pineal gland at night or under dark environments, plays a vital role in regulating circadian rhythm, particularly the sleep/wake cycle (Kamfar et al., 2024; Poza et al., 2022) (Figure 1). Under healthy physiological conditions, melatonin is released into systemic circulation and other body fluids in the mid-to-late evening, approximately 2–3 h before habitual bedtime, remaining elevated throughout the night and typically peaking between 02:00 and 04:00 a.m. Concentration of melatonin subsequently decline during the early morning hours, resulting in a characteristic “mesa-shaped” plasma concentration–time profile (Poza et al., 2022; Seiden and Shah, 2019). Dim-light melatonin onset (DLMO), an indication of the endogenous melatonin secretion in the evening, is considered as the key marker of central circadian phase and internal biological clock alignment. With advancing age or under conditions of circadian misalignment, melatonin secretion may decline and the amplitude of DLMO may be altered, contributing to impaired sleep quality and circadian dysregulation (Nuszkiewicz et al., 2025). Given melatonin's central role in biological rhythms and sleep initiation, demand for synthetic melatonin as a dietary supplement has grown substantially; with global production estimated at approximately 4,000 metric tons in 2019 and projected annual growth exceeding 10% (Zisapel, 2018; Skrzelowski et al., 2021).

**Figure 1:**
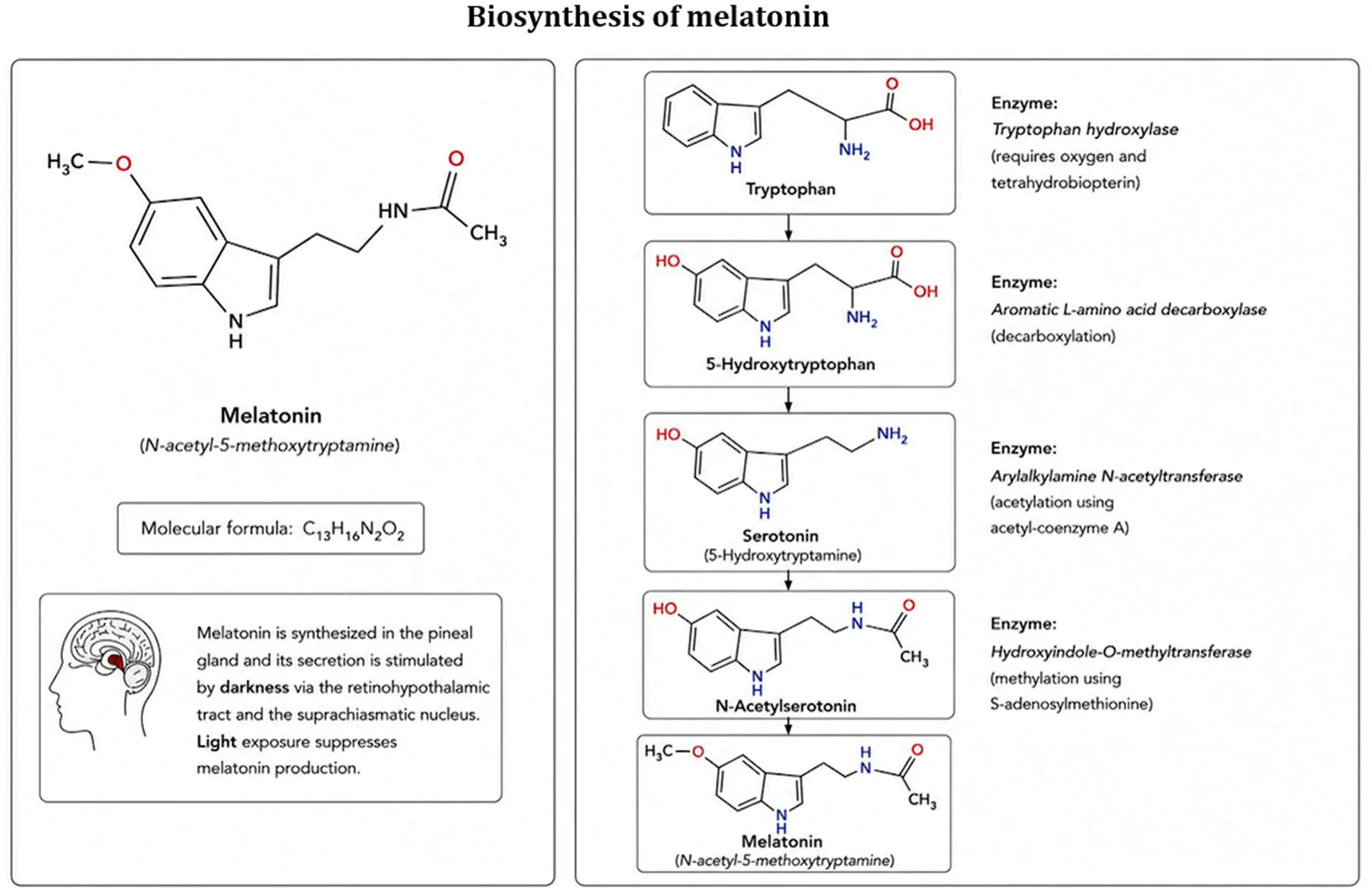
Biosynthesis of melatonin: melatonin is synthesized from the amino acid tryptophan. Tryptophan is converted to 5-hydroxytryptophan by the enzyme tryptophan hydroxylase. 5-Hydroxytryptophan is then converted to serotonin by the enzyme aromatic L-amino acid decarboxylase. Serotonin is subsequently converted to N-acetylserotonin by the enzyme arylalkylamine N-acetyltransferase, the rate-limiting enzyme in melatonin synthesis. Finally, N-acetylserotonin is converted to melatonin by the enzyme acetylserotonin O-methyltransferase.

Despite the widespread use of synthetic melatonin as sleep aid in the United States, Canada, Europe, China, and Australia at doses ranging from 1 to 10 mg, large-scale, long-term randomized controlled trials evaluating its safety in adults and children remain limited (Boutin et al., 2023). Moreover, long-term safety data in individuals with cardiovascular, reproductive, endocrine, and metabolic disorders are scarce, despite reports suggesting potential interactions with prescription medications and disease-specific physiological pathways (Boutin et al., 2023). The health information about the side effects of melatonin as revealed by the National Institute of Health (NIH) includes headaches, dizziness, nausea, palpitations, and daytime sleepiness (Tuft et al., 2023). Notably, regulatory agencies such as the French Agency for Food, Environmental and Occupational Health & Safety (ANSES), along with similar reports from other European countries and Canada, have raised concerns regarding a potential association between melatonin use and sudden infant death syndrome (SIDS), although the evidence remains inconclusive (Shimomura et al., 2019). Importantly, recent observational findings presented at the American Heart Association (AHA) scientific sessions reported the possible link between long-term melatonin usage (for at least 1 year) in insomnia and the higher risk of heart failure diagnosis or hospitalization (Nnadi et al., 2025). Another unresolved concern is the possible suppression of endogenous melatonin synthesis following long-term exogenous supplementation, potentially leading to dependency (Gerdin et al., 2004). In addition, the effect of synthetic melatonin on stress regulation and cortisol dynamics remains inconclusive, with several studies reporting no significant changes even after prolonged supplementation (Pachimsawat et al., 2024).

Recently several herbal extracts such as Valerian, Saffron, Chamomile, Lemon balm, Ashwagandha etc. have emerged as nutraceuticals to improve sleep quality (Oskouei et al., 2018; Ebrahim et al., 2025). Although some of these herbals may modulate neurotransmitter pathways, their ability to enhance endogenous melatonin secretion or restore the cortisol–melatonin balance is limited. Black cumin or black seed (*Nigella sativa*) is a popular medicinal herb and culinary spice widely using in Indian and Arab traditional systems of medicine (Yimer et al., 2019; Rawwash et al., 2025). Recently a proprietary *Nigella sativa* oil extract rich in thymoquinone (registered as ThymoDream ^®^/BlaQmax^®^, hereinafter mentioned as “TD”) has been shown to modulate the hypothalamic–pituitary–adrenal (HPA) axis and improve melatonin concentration within the physiological range in the absence of exogenous melatonin (Saneha et al., 2023; Mohan et al., 2023, 2024). Randomized controlled trials employing both subjective and objective outcome measures have further demonstrated that TD improves the stress–sleep–immunity axis by reducing stress, enhancing sleep quality, and improving immune function (Mohan et al., 2023, 2024). However, no investigation has been reported on its influence on nocturnal plasma melatonin profile, especially in comparison with exogenous melatonin supplementation. Accordingly, the present randomized, double-blind, active-controlled crossover study was designed to compare the effects of a single dose and 7-day repeated dose treatment of TD (200 mg/day) against synthetic melatonin (s-Mel; 5 mg/day) on nocturnal plasma melatonin profile and sleep quality, employing healthy adult volunteers having self-reported non-restorative sleep issues. Sleep quality was evaluated using actigraphy on Days 1, 3, and 7. Daytime sleepiness and functional alertness were assessed using the Epworth Sleepiness Scale (ESS).

## 2 Materials and methods

### 2.1 Study design

The study followed a randomized, double-blind, active-controlled crossover design as schematically illustrated in Figure 2. The protocol was approved by the registered Institutional Ethics Committee and prospectively registered with the Clinical Trial Registry of India (CTRI/2024/11/077454) dated 28/11/2024 and the first participant was enrolled on 09/12/2024, in accordance with the Government of India's clinical research guidelines. Written informed consent was obtained from all participants prior to enrolment, and the study was conducted in accordance with the Declaration of Helsinki and Good Clinical Practice (GCP) guidelines. Crossover design was chosen to minimize inter-individual variability and enhance statistical power while allowing each participant to serve as their own control.

**Figure 2:**
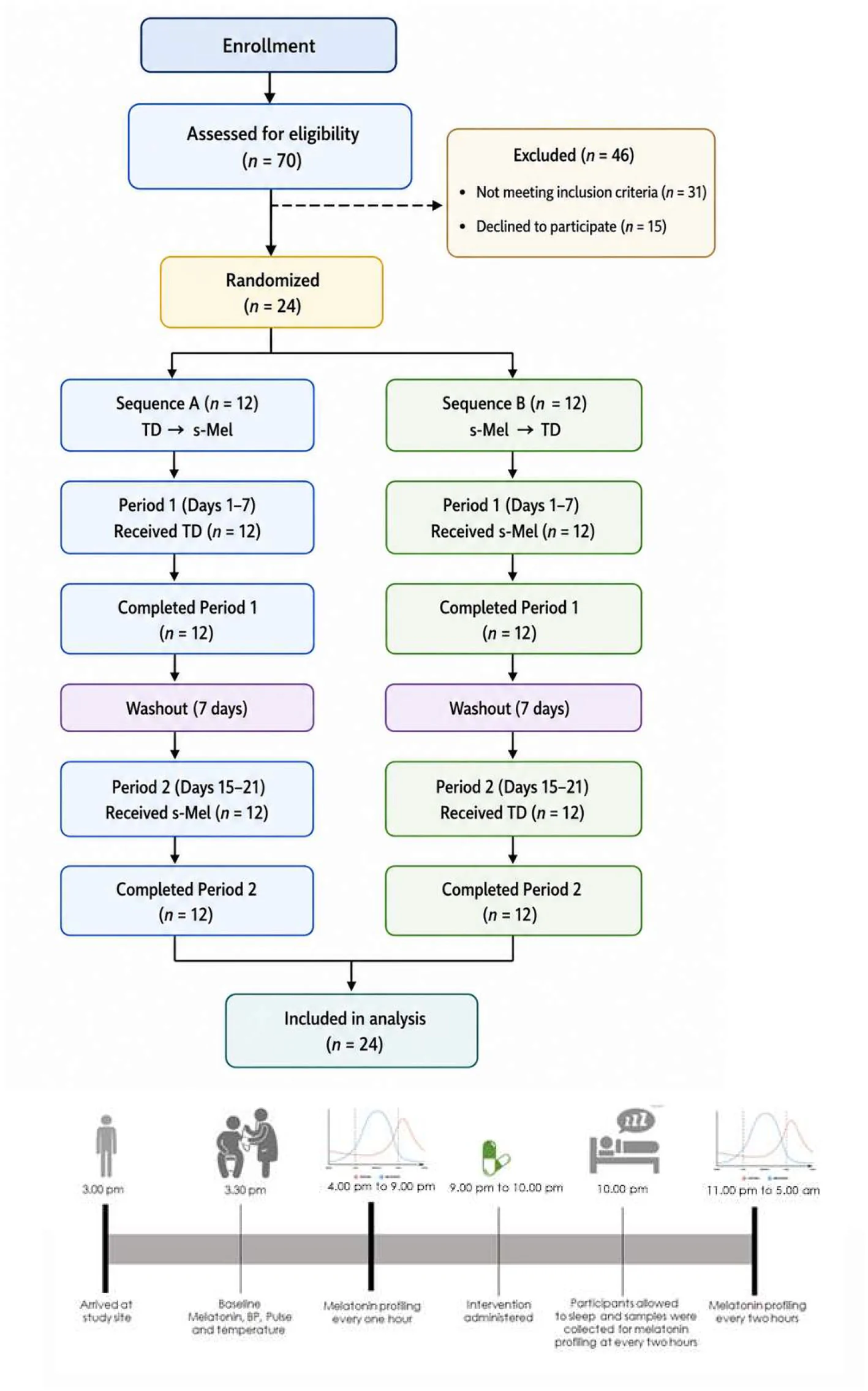
Consort diagram illustrating the study design.

### 2.2 Sample size

An a priori sample size calculation was performed using G^*^Power version 3.1.9.7 for a repeated-measures ANOVA (within–between interaction). A medium effect size (Cohen's *f* = 0.25) was assumed, with a two-sided α level of 0.05 and 80% power. The calculation was based on two groups and 10 repeated measurements, with a correlation of 0.5 among repeated measures and a non-sphericity correction (ε) of 1.0. These assumptions yielded a minimum required sample size of 14 participants. No Bonferroni adjustment was applied. To allow for potential dropouts, the target sample size increased to 24 participants.

### 2.3 Participant recruitment and randomization

The participants were enrolled and assigned through the outpatient facility of Balagangadharanatha Swami Global Institute of Medical Sciences, Bangalore, India and from the volunteer database of Leads Clinical Research and Bio-Services Pvt. Ltd, Bangalore, India. Those who were willing to join the study were screened, based on the inclusion/exclusion criteria, as detailed in Table 1. Eligible participants were randomized in a 1:1 allocation ratio using a computer-generated block randomization method (www.randomization.com). An identification number was assigned to each participant based on the order of enrolment. Randomization codes were generated and maintained by an independent statistician and provided to the investigator in sealed, opaque envelopes to ensure allocation concealment. To minimize the bias, blinding was maintained until all data were sealed for analysis.

**Table 1:** Inclusion and exclusion criteria.

Inclusion criteria	Exclusion criteria
Participants aged 18–70 years	Participants suffering from mental disorders, clinical anxiety, depression, Cushing's syndrome, and other major ailments
Participants with Pittsburgh Sleep Quality Index (PSQI) scores between 5 and 15	Participants on medication/dietary supplements that may affect sleep disorders, anxiety, depression such as prednisone, dexamethasone, and other steroids administered orally or intravenously
Participants with no history of psychiatric conditions and should be ready to abstain from alcohol consumption, smoking, and consumption of caffeinated beverages	Pregnant or lactating women, participants on hormonal birth-control measures or participants allergic to herbal products, nuts or any components of the study product.
Participants who are willing to provide informed consent and ready to adhere to the study procedures.	Participation or recently participated in another clinical trial within the last 3 months prior to the study initiation. Any additional condition(s) at the Investigator's discretion warranting exclusion from or preventing completion of the study.

### 2.4 Intervention and dosage

The proprietary black cumin extract TD and s-Mel were used as investigational products (IPs). TD (IP1) was manufactured from the dried black cumin seeds that were taxonomically identified and authenticated by a qualified botanist, in accordance with Good Manufacturing Practices (Batch No: BCOQ 07/24; Date: 07/09/2024). The thymoquinone (TQ) content was quantified using high-performance liquid chromatography (HPLC) (Shimadzu Analytical India Pvt. Ltd., Mumbai, India), following established protocols (Thomas et al., 2022). Other key bioactive terpenes and terpenoids were identified, confirmed, and quantified using gas chromatography-tandem mass spectrometry (GC–MS/MS) (Shimadzu Analytical India Private Limited, Mumbai, India) as previously mentioned (Das et al., 2022). The analytical standards of TQ (CAS No: 490-91-5) and melatonin (CAS No:73-31-4) were procured from Sigma-Aldrich (Bangalore, India). Synthetic melatonin supplement (IP2) was procured from Nature Made, Southern California, USA (Lot No: 2557471).

A double-dummy design was employed to ensure blinding, as the two treatments (TD as soft gel capsule and s-Mel as a tablet) differed in physical appearance. Matching placebos, identical in size, shape, color, and appearance, were manufactured for both formulations. Airtight high-density polyethylene containers, each containing 15 units [either soft gel capsules of TD containing 200 mg (+10% overage) black cumin extract per capsule or s-Mel tablets containing 5 mg melatonin], along with their respective matching placebo, were distributed to the participants. Each participant received both a soft gel capsule and a tablet daily, one being active and the other a placebo, depending on their randomized group allocation. Participants were instructed to consume IPs after dinner, at 09:30 p.m. and to switch off lights by 10:00 p.m. The total study duration was 21 ± 2 days, including 7 days wash out period. At each visit, the remaining capsules and tablets were recorded to assess the compliance. To evaluate the effectiveness of blinding, participants were asked to guess their treatment allocation.

### 2.5 Outcome measures

#### 2.5.1 Plasma melatonin profiling and DLMO

Plasma melatonin profiling and DLMO were the primary outcomes of the study. Plasma melatonin concentration of 10 pg/mL was considered as the fixed threshold value for DLMO estimation, since DLMO was operationally defined as the time at which plasma melatonin concentration reached and remained above a fixed threshold of 10 pg/mL (Skubic et al., 2025). However, DLMO was not calculated for s-Mel since the observed plasma melatonin levels have a significant contribution from the exogenous melatonin supplemented. In the present study, plasma samples were collected from 05:00 p.m. to 05:00 a.m. (5, 6, 7, 8, 9, 10, 11 p.m. followed by 1, 3, and 5 a.m.). Light conditions prior to sleep were maintained under dim light (< 8 lux), while samples collected from lights off onward were obtained under sleep condition. A peripheral venous catheter was inserted for continuous blood access and maintained with an infusion of heparin sodium diluted in 0.9% NaCl throughout the sampling period to prevent coagulation. Blood samples were collected into EDTA-coated tubes, immediately centrifuged, and the plasma was aliquoted into microtubes. All plasma samples were frozen at −20 °C immediately after processing and subsequently stored at −80 °C until analysis (Glacet et al., 2023).

Plasma melatonin was quantified using ELISA kit method (Catalogue no: E-EL-H2016), following manufacturer's instruction, (Elabscience Biotechnology Co., Limited, Bethesda, USA). The absorbance was read at 450 ± 2 nm employing Varioskan™ LUX multimode microplate reader (Thermo Scientific™, Waltham, MA, USA).

#### 2.5.2 Secondary outcome measures

Secondary outcomes included objective sleep quality assessed by wrist actigraphy, serum cortisol levels, and subjective daytime sleepiness assessment using the Epworth Sleepiness Scale (ESS).

##### 2.5.2.1 Actigraphy

Wrist actigraphy was used to objectively assess sleep-wake patterns. Wristwatch like actigraphy device (MotionWatch 8; CamNtech Ltd., Cambridge, UK) was worn on the non-dominant wrist and continuously recorded the activity levels which were subsequently analyzed using validated algorithms to estimate the sleep parameters such as sleep latency, total sleep duration, wake-after-sleep-onset (WASO) and sleep efficiency. Participants were instructed to wear the actigraphy device continuously for 7 days as per the study protocol (Hendricks et al., 2025).

##### 2.5.2.2 Daytime sleepiness evaluation by ESS

ESS is a widely used self-administered questionnaire designed to assess subjective daytime sleepiness. It evaluates the likelihood of dozing off in eight common daily situations, with each item scored from 0 (never doze) to 3 (high chance of dozing). The total score ranges from 0 to 24. A score < 7 indicates no significant daytime sleepiness, 8–9 suggests average sleepiness, and a score of ≥10 is indicative of excessive daytime sleepiness (Motlaq et al., 2024; Johns, 1991).

### 2.6 Serum cortisol analysis

Serum cortisol concentrations were measured at baseline and at the end of each intervention period (Day 7) using a commercial ELISA kit (Catalogue No: E-EL-0157, Elabscience Biotechnology Co., Texas, USA). Absorbance was measured at 450 ± 2 nm using the Varioskan™ LUX microplate reader.

### 2.7 Statistical analysis

All statistical analyses were performed using IBM SPSS Statistics version 29.0 (IBM Corp., New York, USA). Continuous variables were expressed as mean ± standard deviation (SD) for normally distributed data. Primary outcomes and actigraphy-derived sleep parameters were analyzed using a linear mixed-effects model (LMM) to account for the crossover design, with treatment, period, and sequence included as fixed effects and participant as a random effect, with statistical significance defined as *P* < 0.05. The model adjusted for sequence and period effects as required for the crossover design. The treatment effect was interpreted after adjustment for these design factors, and all primary results are reported with 95% confidence intervals (CI). Wrist actigraphy data (secondary outcome) and cortisol levels were also analyzed using a 2 × 4 RM ANOVA and 2 × 2 RM ANOVA, with Bonferroni correction applied to adjust for multiple comparisons. Demographic and biochemical parameters were analyzed using independent *t*-tests. All tests were two-tailed with statistical significance set at *P* < 0.05.

## 3 Results

### 3.1 Participant disposition and baseline characteristics

A total of 24 participants both males and females (36.2 ± 4.6 years, BMI: 25.3 ± 2.3 kg/m^2^) were enrolled, randomized, and completed both intervention phases, and were therefore included in the final analysis. No participant withdrawals or protocol deviations were reported during the study period. Baseline demographic and clinical characteristics were comparable between the two randomized sequences, indicating successful randomization and absence of selection bias (Table 2).

**Table 2:** Participant profile at baseline.

Parameters	Sequence A (TD → s-Mel) (*n* = 12)	Sequence B (s-Mel → TD) (*n* = 12)
Sex (Male/Female)	4/8	7/5
Age (Years)	36.5 ± 4.2	35.2 ± 5.4
Body weight (Kg)	63.2 ± 8.2	66.5 ± 7.0
BMI (Kg/m^2^)	24.5 ± 1.6	25.0 ± 2.1
Systolic blood pressure (mmHg)	125.7 ± 10.2	120.8 ± 8.7
Diastolic blood pressure (mmHg)	80.5 ± 9.5	76.1 ± 9.0
Pulse rate (beats/min)	78.5 ± 8.0	78.4 ± 7.6

BMI, Body Mass Index. Values are expressed as mean ± SD. A P-value < 0.05 indicates statistical significance.

There were no statistically significant differences in baseline sleep parameters, plasma melatonin levels, or serum cortisol concentrations, confirming homogeneity at study initiation.

### 3.2 Effect of interventions on plasma melatonin profile

Melatonin profiling was carried out for 12 h (from 05:00 p.m. to 05:00 a.m.) and plasma concentration vs. time plot was constructed to understand the influence of TD and s-Mel. Plasma melatonin profiling demonstrated a clear nocturnal rise under dim-light conditions in both intervention phases (*P* < 0.001). Administration of TD resulted in a distinct modulation of the nocturnal melatonin secretion curve compared to s-Mel, as reflected by changes in the timing and magnitude of melatonin elevation (Figures 3a, b).

**Figure 3:**
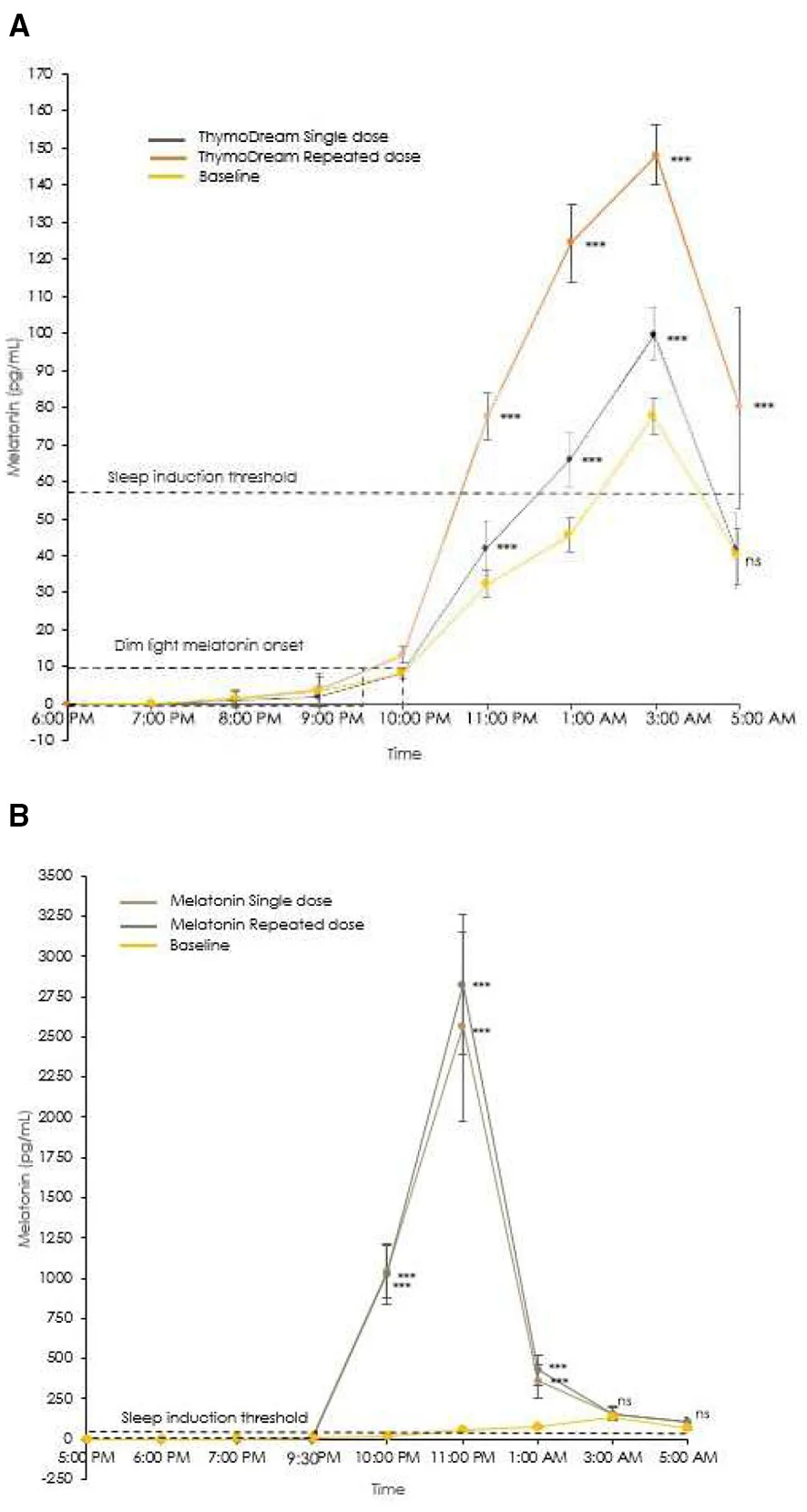
**(a)** Effect of TD on melatonin profile and DLMO. **(b)** Effect of s-Mel on melatonin profile. ***Denotes significance at *p* < 0.001; ns, non significance.

Single dose TD intake elicited an increase in plasma melatonin concentration, from 9.56 ± 1.59 pg/mL at 10:00 p.m. to 114.56 ± 8.05 pg/mL at 03:00 a.m. Notably, the highest melatonin concentration was recorded at 5 h 30 min post-administration (approximately 3:00 a.m.), reaching the peak value and began to decrease after that (Table 3a; Figure 3a). In contrast, s-Mel produced a rapid surge during the first 1 h 30 min, followed by a steady decline in plasma melatonin reflective of its bioavailability. After 3 h 30 min of administration, melatonin concentration dropped markedly and was significantly different from the plasma concentration observed for TD at that point (*P* < 0.001; Table 3a; Figures 3a, b). Seven days of repeated dosing also produced a similar trend with a significant difference in plasma melatonin concentrations between the TD and s-Mel groups for up to 3 h 30 min of post-administration (*P* < 0.001). After 5 h 30 min of administration (03:00 a.m.), plasma melatonin concentrations were no longer significant between the groups (*P* > 0.050). The observed plasma melatonin level at 03:00 a.m. was, TD: 148.06 ± 21.08 pg/mL and s-Mel: 155.36 ± 35.16 pg/mL (*P* = 0.388). After 7 h 30 min of administration (05:00 a.m.) time point, plasma melatonin levels were TD: 79.92 ± 27.01 pg/mL and s-Mel: 106.15 ± 66.55 (*P* = 0.060; Table 3a; Figures 3a, b).

**Table 3a:** Overtime comparison of melatonin profile in TD and s-Mel.

DLMO	Mean ±**SD**				**Intragroup** ***P-value***	
Time	BL	Intervention	SD	RD	TD	s-Mel
10:00 p.m.	7.51 ± 1.14	TD	9.56 ± 1.59	13.35 ± 2.39	< 0.001	< 0.001
		s-Mel	1,040.62 ± 158.99	1,023.62 ± 185.04	< 0.001	< 0.001
11:00 p.m.	30.43 ± 3.52	TD	47.83 ± 8.45	77.65 ± 6.46	< 0.001	< 0.001
		s-Mel	2,565.91 ± 589.30	2,824.99 ± 439.59	< 0.001	< 0.001
01:00 a.m.	42.86 ± 4.29	TD	75.62 ± 8.41	124.26 ± 10.65	< 0.001	< 0.001
		s-Mel	359.70 ± 101.51	428.35 ± 92.44	< 0.001	< 0.001
03:00 a.m.	73.04 ± 4.57	TD	114.56 ± 8.05	148.06 ± 21.08	< 0.001	< 0.001
		s-Mel	160.07 ± 41.02	155.36 ± 35.16	< 0.001	< 0.001
05:00 a.m.	37.38 ± 7.00	TD	47.41 ± 11.63	79.92 ± 27.01	0.003	< 0.001
		s-Mel	110.93 ± 20.75	106.15 ± 66.55	< 0.001	< 0.001

DLMO, Dim light melatonin onset; TD, ThymoDream; s-Mel, synthetic melatonin; BL-Baseline, SD, Single dose; RD, Repeated dose. A P-value < 0.05 indicates statistical significance.

Within treatment overtime analysis at different time points also revealed significant increase of plasma melatonin concentrations at all time points after intake of a single dose of TD compared to the corresponding baseline values (*P* < 0.050). The observed enhancements after 1 h 30 min, 3 h 30 min, 5 h 30 min, and 7 h 30 min of post-administration were 27.3%, 57.2%, 76.4%, 56.8%, and 26.8%, respectively compared to baseline values (Table 3a; Figure 3a). Following repeated dosing of TD for 7 days, plasma melatonin concentrations exhibited further increase at all post- administration time points compared to baseline. The observed increase was 77.8%, 155.2%, 189.9%, 102.7%, and 113.8%, respectively, and were significant with respect to the baseline (*P* < 0.001), and with respect to the respective increase observed with single dose treatment (*P* < 0.001; Table 3a; Figure 3a).

Similarly, single dose administration of s-Mel also demonstrated significant elevation in plasma melatonin concentrations at every time point relative to baseline. s-Mel caused a steady increase in plasma melatonin, [approximately 138.5- fold increase after 30 min of administration (from 7.51 ± 1.14 to 1040.62 ± 158.99 pg/mL)] and decreased sharply to 160.07 pg/mL after 5 h 30 min (~03:00 a.m.). At about 05:00 a.m., the observed plasma concentration was only 110.93 pg/mL (Table 3a; Figure 3b). Seven days of repeated supplementation of s-Mel also caused increase in the plasma concentration, significantly from the baseline (*P* < 0.001). The observed enhancement across the various post-administration time points were respectively 136.3-fold, 92.8-fold, 9.9-fold, 2.1-fold, and 2.84-fold (Table 3a; Figure 3b). However, there was no significant difference between the single dose and 7 days repeated dose treatment (Table 3a; Figure 3b).

Melatonin profile followed by single and repeated dose administration was analyzed using LMM. Single dose demonstrated a significant treatment effect favoring melatonin over TD, [*F*_(1, 198)_ = 949.420, *P* < 0.001], with an estimated treatment difference of 788.45 pg/mL (95% CI: 737.986, 838.907). A significant time effect was also observed, [*F*_(4, 198)_ = 314.336, *P* < 0.001], indicating significant changes in melatonin concentrations across the sampling time points. However, there were no significant effects for sequence (*P* = 0.868) or period (*P* = 0.788; Table 3b). Further, repeated dose administration demonstrated no significant treatment effect [*F*_(1, 220)_ = 0.944, *P* = 0.332], with an estimated treatment difference of −19.565 pg/mL (95% CI: −59.24, 20.11), however, a significant time effect was observed, [*F*_(4, 220)_ = 613.011, *P* < 0.001]. Further, period effect was also significant (*P* < 0.001), while no significant sequence effect (*P* = 0.386) was noted (Table 3b).

**Table 3b:** Melatonin profile following single-dose and repeated-dose administration using LMM.

**Outcome**	Treatment effect		Time effect		Sequence effect	Period effect
	***F-value***	***P-value***	***F-value***	***P-value***	***P-value***	***P-value***
Melatonin Profile-SD	949.420	< 0.001	314.336	< 0.001	0.868	0.788
Melatonin Profile-RD	0.944	0.332	613.011	< 0.001	0.386	< 0.001

LMM, linear mixed-effects model; SD, single dose; RD, repeated dose. P < 0.05 was considered statistically significant.

### 3.3 Influence of TD on DLMO

At baseline, the average predefined plasma melatonin threshold of 10 pg/mL at dim-light condition (DLMO) across the participants were observed at 11:00 p.m. Following the administration of a single dose of TD, the mean DLMO was shifted to 10:00 p.m. Upon repeated administration for seven consecutive days, the DLMO continued to advance progressively to ~09:30 p.m. Upon s-Mel administration, plasma concentration of 10 pg/mL was attained after 60 min of post-administration; however, this is interpreted as the contribution from both endogenous secretion and the bioavailable fraction from the administered melatonin (Figures 3a, b).

### 3.4 Actigraphy-based sleep parameters

Actigraphy data following the supplementation on days 1, 3, and 7 were analyzed by 2 × 4 mixed RM ANOVA to assess the influence of the intervention over time and treatment. Both test substances provided a significant improvement in sleep quality (as assessed by sleep latency, WASO, total sleep time, and sleep efficiency) from the baseline (Figure 4).

**Figure 4:**
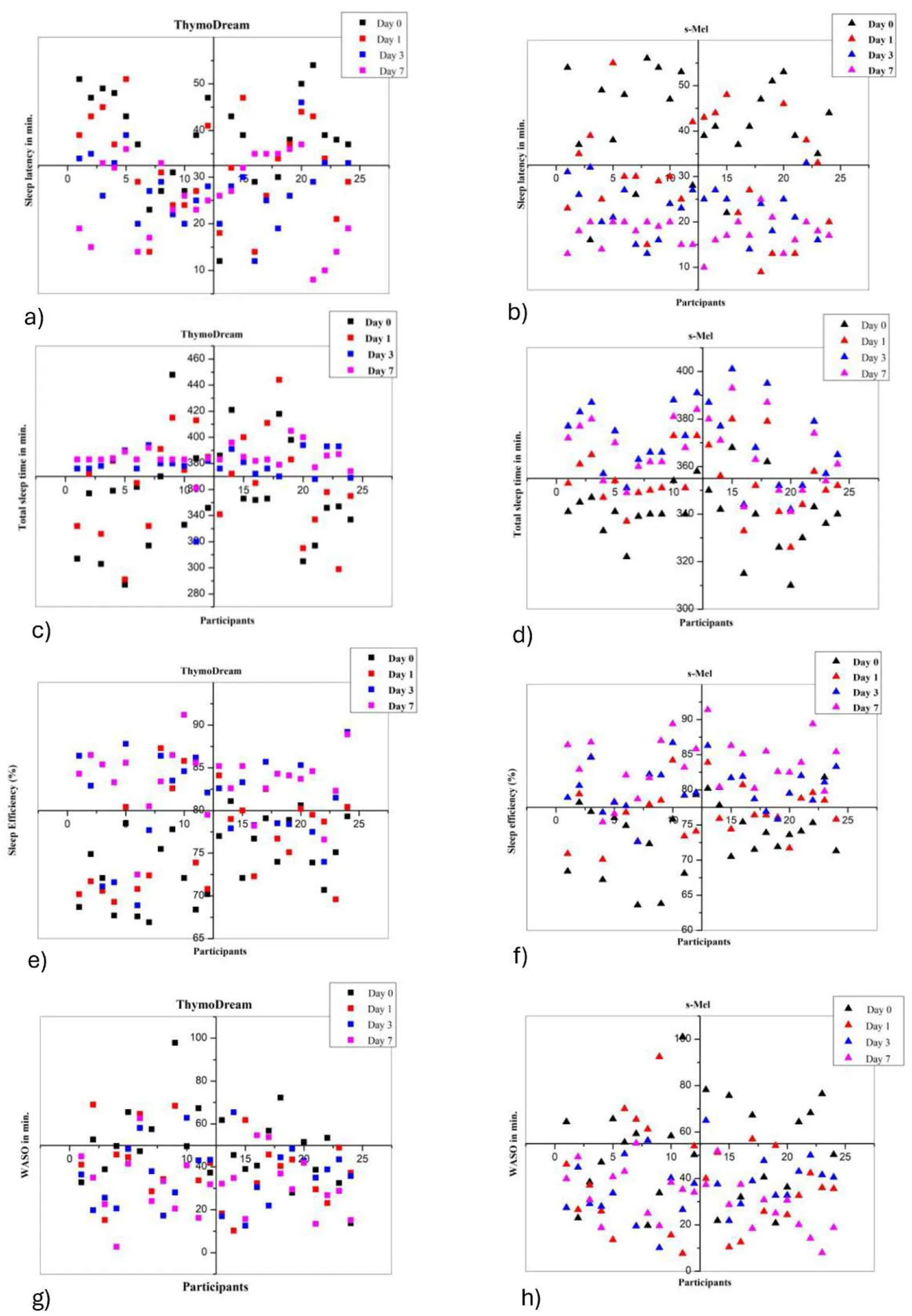
Scatterplot results showing changes in sleep parameters assessed by actigraphy upon treatment with TD and s-Mel on day 0, day1, day 3, and day 7. Sleep latency **(a, b)**; TST **(c, d)**; Sleep efficiency **(e, f)**; WASO **(g, h)**.

Sleep latency showed significant decrease in treatment, time and treatment × time effects upon both TD and s-Mel supplementation. The observed treatment × time effect was [*F*_(3, 69)_ = 3.962, *P* = 0.011, partial η^2^ = 0.147 (95% CI: 21.565, 29.269)]. The main effect of treatment was [*F*_(1, 23)_ = 4.666, *P* = 0.041, partial η^2^ = 0.169 (95% CI: 0.119, 5.526)] and the time effect was [*F*_(3, 69)_ = 41.026, *P* < 0.001, partial η^2^ = 0.641 (95% CI: 12.893, 23.523)] (Table 4a).

**Table 4a:** Outcomes of actigraphy parameters using 2 × 4 Mixed RM ANOVA.

Actigraphy	Effects	Effect size	*F-value*	*P-value*
Sleep latency	Treatment	0.169	4.666	0.041
	Time	0.641	41.026	< 0.001
	Treatment vs. time	0.147	3.962	0.011
WASO	Treatment	0.004	0.102	0.752
	Time	0.296	9.655	0.001
	Treatment vs. time	0.008	0.189	0.904
Total sleep time	Treatment	0.294	9.564	0.005
	Time	0.511	24.059	< 0.001
	Treatment vs. time	0.690	0.629	< 0.001
Sleep efficiency	Treatment	0.003	0.069	0.795
	Time	0.733	63.067	0.001
	Treatment vs. time	0.019	0.439	0.726

A P-value < 0.05 indicates statistical significance.

Analysis of WASO indicated no significant difference with respect to treatment and treatment × time. The treatment × time interaction effect size was [*F*_(3, 69)_ = 0.189, *P* = 0.904, partial η^2^ = 0.008 (95% CI: 25.159, 37.708)], treatment effect size was [*F*_(1, 23)_ = 0.102, *P* = 0.752, partial η^2^ = 0.004 (95% CI: −4.855, 3.556)]. However, a significant difference was observed in time interaction [*F*_(3, 69)_ = 9.655, *P* < 0.001, partial η^2^ = 0.296 (95% CI: 7.681, 30.119)] (Table 4a).

For total sleep time, a significant increase in treatment, time and treatment × time interaction effects were noted. The observed effect on treatment × time was [*F*_(3, 69)_ = 0.629, *P* < 0.001, partial η^2^ = 0.690 (95% CI: 380.919, 388.081)], treatment was [*F*_(1, 23)_ = 9.564, *P* = 0.005, partial η^2^ = 0.294 (95% CI: 4.297, 21.661)] and the time effect was [*F*_(3, 69)_ = 24.059, *P* < 0.001, partial η^2^ = 0.511 (95% CI: −40.537, −15.546)] (Table 4a).

The observed treatment × time interaction [*F*_(3, 69)_ = 0.439, *P* = 0.726, partial η^2^ = 0.019, (95% CI: 81.789, 85.094)] and the treatment effect [*F*_(1, 23)_ = 0.069, *P* = 0.795, partial η^2^ = 0.003 (95% CI: −1.583, 2.044)] were not significantly different for sleep efficiency across both treatments, but the main time effect [*F*_(3, 69)_ = 63.067, *P* < 0.001, partial η^2^ = 0.733 (95% CI: −12.389, 27.211)] was significant (Table 4a).

Considering the sequence, period, treatment and time as fixed effects, LMM analysis was employed for sleep parameters. For sleep latency, a significant treatment effect was observed. TD was associated with a mean increase of 2.8 min in latency compared to the s- Mel [*F*_(1, 161)_ = 5.224, *P* = 0.024; 95% CI: 0.384, 5.262]. A significant period and sequence effect was observed for sleep latency (*P* = 0.024). Additionally, a significant time effect was also observed [*F*_(3, 161)_ = 42.453; *P* < 0.001; Table 4b).

**Table 4b:** Outcomes of actigraphy-derived sleep parameters using LMM.

Actigraphy parameters	Treatment effect		Time effect		Sequence effect	Period effect
	***F-value***	***P-value***	***F-value***	***P-value***	***P-value***	***P-value***
Sleep latency	5.224	0.024	42.453	< 0.001	0.024	0.024
WASO	0.063	0.802	11.274	< 0.001	0.802	0.802
Total sleep time	17.862	< 0.001	19.775	< 0.001	< 0.001	< 0.001
Sleep efficiency	0.175	0.677	59.941	< 0.001	0.677	0.677

LMM, linear mixed-effects model; WASO, wake after sleep onset. P < 0.05 was considered statistically significant.

The analysis of WASO did not reveal a significant treatment effect. The mean difference between the TD and s-Mel was −0.593 [*F*_(1, 161)_ = 0.063, *P* = 0.802; 95% CI: −5.249, 4.063]. Significant time effect was noted for WASO [*F*_(3, 161)_ = 11.274; *P* < 0.001)], whereas no sequence and period effect were observed (*P* = 0.802; Table 4b).

TST demonstrated significant treatment and time effects. TD administration was associated with a mean increase of 12.6 min in comparison with s- Mel; treatment effect size was [*F*_(1, 161)_ = 17.862, *P* < 0.001; 95% CI: 6.748, 18.585], and the time effect was [*F*_(3, 161)_ = 19.775; *P* < 0.001)], with a significant sequence and period effects (*P* < 0.001; Table 4b).

No significant treatment effect on sleep efficiency was noted. TD resulted in a mean difference of 0.23 compared to the s-Mel treatment [*F*_(1, 161)_ = 0.175, *P* = 0.677; 95% CI: −0.858, 1.318]. However, a significant time effect was noted [*F*_(3, 161)_ = 59.941; *P* < 0.001)]. However, no significant period and sequence effects were observed for sleep efficiency (*P* = 0.677; Table 4b).

### 3.5 Subjective daytime sleepiness (Epworth Sleepiness Scale)

Among the 24 participants included in the crossover analysis, the linear mixed-model analysis demonstrated a significant treatment effect in TD over melatonin, [*F*_(1, 69)_ = 13.428, *P* < 0.001; 95% CI: 9.390, 10.110], with an estimated treatment difference of −0.708 (95% CI: −1.094, −0.323). A significant time effect was also observed [*F*_(1, 69)_ = 24.580, *P* < 0.001]. Sequence and period effects were also significant (*P* < 0.001; Table 5).

**Table 5:** 

Outcome	Treatment effect		Time effect		Sequence effect	Period effect
	***F-value***	***P-value***	***F-value***	***P-value***	***P-value***	***P-value***
ESS (*n* = 24)	13.428	< 0.001	24.580	< 0.001	< 0.001	< 0.001
ESS (*n* = 9)	11.918	0.002	39.629	< 0.001	0.002	0.002

LMM, linear mixed-effects model; ESS, Epworth Sleepiness Scale. P < 0.05 was considered statistically significant.

However, detailed analysis of the individual scores showed that only 9 participants (out of 24 enrolled) were experiencing significant daytime sleepiness at the baseline (ESS score >11). A significant treatment effect in TD [*F*_(1, 24)_ = 11.918, *P* = 0.002], with a treatment difference of −0.944 (95% CI: −1.509, −0.380) was observed. A significant time effect was also noted [*F*_(1, 24)_ = 39.629, *P* < 0.001]. Sequence and period effect were also significant (*P* = 0.002; Table 5).

### 3.6 Influence of TD on serum cortisol levels

The effect of TD on serum cortisol level was evaluated using 2 × 2 RM ANOVA. A significant treatment × time interaction [*F*_(1, 24)_ = 103.75, *P* < 0.001, partial η^2^ = 0.819 (95% CI: 18.073, 21.569)] was observed in TD compared to s-Mel. Though the main effect of treatment was not significant [*F*_(1, 24)_ = 1.641, *P* = 0.213, partial η^2^ = 0.067 (95% CI: −1.978, 0.465)], the time effect was significant for TD [*F*_(1, 24)_ = 48.914, *P* < 0.001, partial η^2^ = 0.680 (95% CI: 1.706, 3.140)] (Table 6a).

**Table 6a:** Serum cortisol analysis using 2 × 2 RM ANOVA.

Outcome	Groups	**Mean** ±SD		Effects	Effect size	*F-value*	*P-value*
		**Baseline**	**Day 7**				
Cortisol (μg/dL)				Treatment	0.067	1.641	0.213
	TD	25.22 ± 4.75	19.82 ± 4.13	Time	0.680	48.914	< 0.001
	S-Mel	23.00 ± 5.38	23.55 ± 5.09	Treatment vs. time	0.819	103.758	< 0.001

Values are expressed as mean ± SD. A P value < 0.05 indicates statistical significance.

LMM analysis of cortisol did not demonstrate a significant treatment effect between TD and s-Mel [*F*_(1, 69)_ = 3.096, *P* = 0.083], with an estimated treatment difference of −0.756 (95% CI: −1.614, 0.101). However, a significant time effect was observed [*F*_(1, 69)_ = 31.777; *P* < 0.001]. No significant sequence and period effects were noted (*P* = 0.083; Table 6b).

**Table 6b:** Serum cortisol analysis using LMM.

Outcome	Treatment effect		Time effect		Sequence effect	Period effect
	***F-value***	***P-value***	***F-value***	***P-value***	***P-value***	***P-value***
Cortisol	3.096	0.083	31.777	< 0.001	0.083	0.083

LMM, linear mixed-effects model. P < 0.05 was considered statistically significant.

### 3.7 Adverse events

Both TD and s-Mel were well tolerated, with no significant adverse effects. However, both the number of subjects and severity of discomforts were significantly higher among s-Mel participants. About 10 participants reported adverse events during treatment with s-Mel, including palpitation (*n* = 2), light headedness (*n* = 5), daytime sleepiness (*n* = 9), headache (*n* = 2), and gastrointestinal discomforts (*n* = 4). The adverse events observed in TD were mainly not related to the intervention, such as bloating (*n* = 2) and flu-like symptoms (*n* = 1). These symptoms were self-limiting and did not necessitate discontinuation.

## 4 Discussion

The present randomized, double-blind, cross-over study evaluated the influence of TD (200 mg/day) in comparison with s-Mel (5 mg/day) on plasma melatonin concentration and sleep quality among healthy participants reported with non-restorative sleep concerns (PSQI score: 5–15). The study employed actigraphy to assess the quality of sleep and compared the data on days 1, 3, and 7, corresponding to single-dose, 3-day, and 7-day supplementation of the IPs. The importance of this protocol also lies in the fact that even short-term sleep deprivation has been shown to trigger widespread adverse effects on overall health and wellbeing (Medic et al., 2017). A linear mixed model analysis was performed to evaluate sequence and period effects. No significant sequence or period effects were observed for single-dose melatonin profiling, sleep efficiency, or wake after sleep onset, supporting the validity of the crossover design for these outcomes. In contrast, significant sequence and period effects were observed during repeated-dose melatonin profiling, as well as for sleep latency and total sleep time, suggesting treatment order- and period-related influences following repeated administration without affecting the overall interpretation of the study findings. The sequence and period effects observed in the current study may be partly defined as derived from TD rather than s-Mel. Usually intake of oral melatonin (1–5 mg), allows melatonin to peak within an hour of ingestion and return to basal concentrations within 4 to 8 h (Tordjman et al., 2017). A pharmacokinetic crossover study reported elimination half-life of ~ 0.95 h for melatonin, with plasma melatonin concentrations declining to low levels within a few hours after administration (Mun et al., 2024). Therefore, the 7-day washout period used in the present study is substantially longer than that required for complete elimination of exogenous melatonin.

Melatonin profiling (through 05:00 p.m. to 05:00 a.m.) and hence the DLMO shift was also estimated. We employed the most widely used fixed-threshold method which defines DLMO as the time at which interpolated melatonin concentration reaches 10 pg/mL in plasma or 3–4 pg/mL in saliva (Skubic et al., 2025). Both TD and s-Mel produced clinically meaningful improvements in nocturnal plasma melatonin profiles and sleep outcomes. The pattern and duration of melatonin modulation and the observed reduction in cortisol levels by two interventions highlight a fundamentally different and more physiologically aligned approach to sleep support by TD: as a natural plant-based agent to enhance sleep by working in harmony with the body's endogenous neuroendocrine pathways rather than introducing supraphysiological levels of bioidentical melatonin.

Melatonin profiles demonstrated that both s-Mel and TD produced significant increase in plasma melatonin concentration across the nocturnal sampling period. However, the melatonin profiles of the TD and s-Mel were distinctly different. While TD caused a progressive and sustained pattern potentially offering a prolonged elevation of melatonin levels in systemic circulation, the rapid elevation that s-Mel produced in the initial couple of hours followed by a sharper decline was consistent with previously reported pharmacokinetics of melatonin (Andersen et al., 2016). The sustained melatonin availability with TD suggests enhanced circadian support rather than transient pharmacological replacement. However, the observed plasma melatonin concentration at 03:00 a.m. and 05:00 a.m. for s-Mel were not statistically different from the observed levels upon repeated dose treatments with TD (*P* > 0.050). These findings suggest that melatonin levels were sustained for ~6 h upon TD administration supporting earlier threshold attainment through modulation of the endogenous melatonin rhythm, unlike s-Mel which impose supraphysiological peaks due to the absorption/bioavailability of s-Mel.

DLMO is considered as a marker of central circadian phase, and its advancement is directly associated with improved sleep initiation and morning alertness (Kennaway, 2023). At the baseline, melatonin concentrations entered the sleep-initiation range (30–45 pg/mL) by approximately 11:00 p.m. in both TD and s-Mel groups; higher nocturnal concentrations were sustained later in the night, around 01:00 a.m. Administration of a repeated dose of TD produced significant DLMO shift to 10:00 p.m., which further shifted to 09:30 p.m. after 7 days of continuous dosing. This indicates that TD attained the onset of melatonin secretion by approximately 60 min following a single dose, and 90 min following 7 days of repeated dosing, demonstrating the potential of TD to regulate the circadian axis. However, the observed reduction in time to attain 10 pg/mL of plasma melatonin concentration, upon both single and repeated dose of s-Mel, was not considered as DLMO shift since the measured plasma melatonin concentration included the contribution from exogenous melatonin as well.

Objective sleep quality was assessed using actigraphy. While polysomnography (PSG) is considered the gold standard for measuring sleep parameters, actigraphy provides a validated and less intrusive alternative to PSG, allowing an easy and reliable assessment of sleep patterns (Vallières and Morin, 2003). The present study demonstrated that TD, even after a single dose, was associated with improvements in sleep latency and total sleep time. Sleep efficiency progressively improved after 3 and 7 days of continuous supplementation. By day 7, outcomes with TD were comparable to s-Mel, with no statistically significant differences in sleep efficiency or WASO; however, sleep latency and total sleep time differed significantly between interventions. These findings agree with the prior results highlighting the sleep-improving properties of thymoquinone-rich black cumin extract against placebo upon 7 days of supplementation (Mohan et al., 2024).

Daytime sleepiness is a frequently observed adverse event with melatonin and other sleep aids and drugs. In this study, we used the validated ESS questionnaire to examine the influence of TD on daytime sleepiness, particularly in comparison with s-Mel. ESS has already been used in short-term intervention studies to monitor changes in daytime sleepiness during the intervals of 7 days (Maurer et al., 2022). However, it has been advised to interpret the data cautiously, because ESS captures habitual sleep propensity over recent weeks. When analyzing the whole 24 participants together, we observed a significant reduction in ESS scores following TD intake (14.5%; *P* < 0.001). A detailed individual data, however, revealed that only 9 out of the 24 participants reported clinically relevant daytime sleepiness at baseline (ESS score > 11). For these individuals, 7 days of TD supplementation led to a marked 25.7% decrease in ESS scores, while s-Mel treatment produced no significant effect. It was reported that daytime sleepiness risk appears to increase at doses ≥ 2 mg of melatonin, becomes more noticeable around 3–5 mg, and is more clearly elevated at doses >10 mg (Tuft et al., 2023).

Significant reduction of cortisol levels upon TD supplementation is an important finding of the study indicating a plausible mechanistic reason for the improvement in sleep quality. Hyperarousal is one of the most important pathophysiological concepts in insomnia research (Dressle et al., 2022). Acute stressors may activate the hypothalamic–pituitary–adrenal (HPA) axis, increasing corticotropin-releasing hormone (CRH), which can cause adrenocorticotropic hormone (ACTH) and cortisol secretion leading to the difficulty in sleep initiation and further sleep fragmentation (Dressle et al., 2022; Riemann et al., 2010). Orexin/Hypocretin is a neuropeptide that plays a central role in regulating arousal and wakefulness by activating adrenal cortisol release (Hirotsu et al., 2015; Bonnavion et al., 2016). Previous clinical study reported a reduction in orexin and cortisol when treated with TD without affecting daytime activities (Saneha et al., 2023; Mohan et al., 2023). Thus, it may be concluded that TD strengthens the circadian rhythm as evident from the DLMO shift and alleviates stress-related hormonal activity; thereby creates a neuroendocrine environment that is more conducive to restorative sleep, whereas s-Mel which helps sleep only through its bioavailability and hence the ability to increase plasma melatonin to supraphysiological levels for 2 to 3 h post-administration (Burgess et al., 2017). This is again the reason for the recommendation that individual's DLMO must be evaluated, and melatonin administration has then to be personalized at optimal time for efficacy (Swanson et al., 2024), while TD can be administered just 30 min prior to bedtime. It was shown that the melatonin supplementation at 2 to 3 h prior to the habitual sleep onset yields only modest effects.

Another consideration regarding melatonin supplementation is the development of tolerance or receptor desensitization over time. Melatonin acts primarily through two G protein-coupled receptors (GPCRs), MT1 and MT2, which are widely distributed in mammalian tissues, particularly in the suprachiasmatic nucleus (SCN) of the hypothalamus. When exposed to supraphysiological concentrations of melatonin, MT2 receptors rapidly desensitize and become less responsive, while MT1 receptors, although more resistant, lose sensitivity after prolonged exposure. Such receptor desensitization may attenuate responsiveness to endogenous melatonin, potentially disrupting natural circadian regulation (Gerdin et al., 2004). So, care must be taken on dosage and duration of melatonin intake, especially the bioavailability enhanced and sustained release formulations of exogeneous melatonin (Braam et al., 2010).

Despite the novelty and encouraging results, some of the limitations of the present study should be acknowledged. First, the relatively short intervention period and modest sample size which may restrict the generalizability of the results, particularly for long-term use or in larger, more diverse populations. Second, age-related differences in melatonin secretion and circadian timing also remain a potential source of heterogeneity. Further, as the participants were healthy adults with mild to moderate sleep complaints, the outcomes may not directly translate to individuals with clinical insomnia, psychiatric comorbidities, or chronic medical conditions. In addition, potential lifestyle and environmental confounders, such as diet, caffeine intake, screen exposure, and physical activity, were not tightly controlled and may have influenced sleep outcomes. Finally, while cortisol and melatonin were assessed as key biomarkers in the present study, future investigations should incorporate broader physiological markers such as orexin, inflammatory cytokines, salivary cortisol and melatonin, urinary 6-sulfatoxymelatonin (aMT6s), and clock gene expression, to better delineate the mechanisms underlying the observed effects.

## 5 Conclusion

The present randomized, double-blind, active-controlled cross-over study evaluated the comparative effects of a black cumin oil extract (TD) and synthetic melatonin (s-Mel) on plasma melatonin profile, sleep quality, and cortisol level in healthy volunteers characterized with mild to moderate non-restorative sleep problems. During 7 days of treatment, TD produced a sustained increase in nocturnal melatonin concentrations over ~6 h of post-administration period with a progressive earlier threshold attainment in DLMO. Unlike s-Mel, which elicited a rapid but transient rise in plasma melatonin, TD supported physiological endogenous melatonin modulation. The observed melatonin levels were not significantly different at about 03:00 and 05:00 a.m. following TD and s-Mel treatments, indicating a favorable sleep balancing effect of TD rather than reliance on an external chronobiotic signal. Though the underlying mechanisms and hormonal profiles differed between TD and s-Mel, both improved the key actigraphy sleep parameters including sleep latency, WASO, total sleep time, and sleep efficiency, particularly after seven days of repeated dosing. Importantly, TD significantly reduced cortisol levels, an effect not generally observed for melatonin supplements. Collectively these findings suggest that TD may act as a natural alternative to conventional melatonin for improving sleep quality and circadian rhythm alignment.

## Data Availability

The original contributions presented in the study are included in the article/supplementary material, further inquiries can be directed to the corresponding authors.
